# The association of chronotype, sleep duration and trajectories of health-risk behaviors among college students: a cohort study

**DOI:** 10.1186/s13034-025-00861-0

**Published:** 2025-01-27

**Authors:** Wanyu Che, Chenfang Wang, Shuman Tao, Tingting Li, Yang Xie, Fangbiao Tao, Xiaoyan Wu

**Affiliations:** 1 Department of Maternal, Child and Adolescent Health, School of Public Health, Anhui Medical University, No. 81 Meishan Road, Hefei, 230032 Anhui China; 2 MOE Key Laboratory of Population Health Across Life Cycle, No 81 Meishan Road, Hefei, 230032 Anhui China; 3 NHC Key Laboratory of Study on Abnormal Gametes and Reproductive Tract, No 81 Meishan Road, Hefei, 230032 Anhui China; 4 Anhui Provincial Key Laboratory of Environment and Population Health across the Life Course, Anhui Medical University, No 81 Meishan Road, Hefei, 230032 Anhui China; 5 Ma’anshan Maternal and Child Health Hospital of Anhui Province, Maanshan, Anhui China

**Keywords:** Chronotype, Sleep duration, Health-risk behaviors, Latent class growth analysis

## Abstract

**Purpose:**

To describe the trajectories of health-risk behaviors (HRBs) among college students through four consecutive surveys and explore the relationship between chronotype, sleep duration and different trajectories of HRBs.

**Methods:**

We used a data sample of 1,042 college students from the College Student Behavior and Health Cohort Study. Students reported sleep parameters, including chronotype (Morningness-Eveningness Questionnaire-5, MEQ-5) and sleep duration. The behavior scale was used to evaluate four HRBs (smoking, alcohol use, low physical activity, smartphone addiction). The latent class growth analysis (LCGA) was used to estimate the trajectory of self-reported HRBs. Multivariate logistic regression models were used to study whether sleep parameters (chronotype and sleep duration) correlated with HRBs^’^ trajectories.

**Results:**

Four unique trajectories of behaviors were identified: unhealthy group (7.4%), increasing group (21.3%), decreasing group (10.3%) and healthy group (61.0%). Compared with the normal sleep, results from logistic regression analyses indicated that long sleep (> 9 h) was associated with the decreasing group and the unhealthy group (*P* < 0.05), while short sleep (< 7 h) was associated with the increasing group and the unhealthy group (*P* < 0.05). Compared with the M-type, the E-type were positively correlated with the unhealthy group, the increasing group, and the decreasing group (*P* < 0.05).

**Conclusion:**

E-type, short sleep duration and long sleep duration were significantly associated with the trajectory of HRBs. Findings underscore the need for targeted screening and prevention of modifiable sleep behaviors with the aim of improving HRBs in college students.

## Introduction

Lifestyle behaviors are leading contributors to global disease burden. Among the top 20 risk factors are smoking, alcohol, drug use, and poor diet [1]. HRBs, such as smoking, alcohol consumption, and physical inactivity often accumulate during adolescence and are relatively persistent throughout the life course [2]. Unfortunately, many adolescents do not meet the guidelines for specific health behaviors, prevalence rates for unhealthy behaviors among adolescents vary from 5.0 to 88.5% [3]. HRBs contribute to the leading causes of mobility and mortality among youth and adults, which cause serious public health problems [4]. In addition, HRBs are also environmental risk factors leading to chronic noncommunicable diseases [5].

Research shows that HRBs commonly occur at the same time or cluster together [6, 7], rather than being isolated from other HRBs that occur alone. For example, adolescents who smoke regularly have also been found to engage in high alcohol consumption and risky sexual behavior [8]. Using data from the Health Survey for England, the research found that in 2008, the proportion of men with four multiple risk behaviors in the 16–24 age group was 6.7%, and the proportion of women was 5.7% [9]. Some studies try to describe the changes in HRBs with the age of adolescents [10], but most trajectory models focus on describing a single risk behavior. According to the risk accumulation hypothesis, exposure to multiple HRBs may lead to long-term health disadvantages [11], highlighting the need for greater knowledge about developmental trajectories of HRBs.

College life represents an important transition period for young adults characterized by increased social involvement. During this critical period, lifestyles acquired will affect the future life of young college students [12]. Chronotype is used to reflect the preference of the individual’s internal circadian rhythm and activity time [13]. Generally, it is divided into three types: morning-type (M-type), neutral-type (N-type) and evening-type (E-type) through questionnaires [14]. The M-type usually shows early sleep and early rise, the E-type usually shows late sleep and late rise, and the one in between is called the N-type. Sleep health is a multidimensional concept that refers to sleep duration, sleep continuity, and quality or satisfaction [15]. E-type, short sleep duration and long sleep duration are common sleep problems among college students [16–18]. Recent studies have discovered that chronotype and sleep duration are associated with adolescent alcohol use, low physical activity, and mobile phone use [19–21]. Unfortunately, most of these studies are cross-sectional. Multiple sleep parameters, including chronotype and sleep duration, are associated with HRBs, suggesting different aspects of disrupted sleep health affect HRBs in adolescents. Despite the increasing number of studies on HRBs, few studies have explored the impact of different sleep dimensions on the developmental trajectories of multiple HRBs, especially among adolescents.

We hypothesize that sleep parameters are associated with multiple trajectories of HRBs. We carried out an epidemiological survey among college students, with the aim of exploring the trajectories of HRBs and examining the prospective associations between chronotype, sleep duration and the trajectories of HRBs.

## Materials and methods

### Participants

**Fig. 1:**
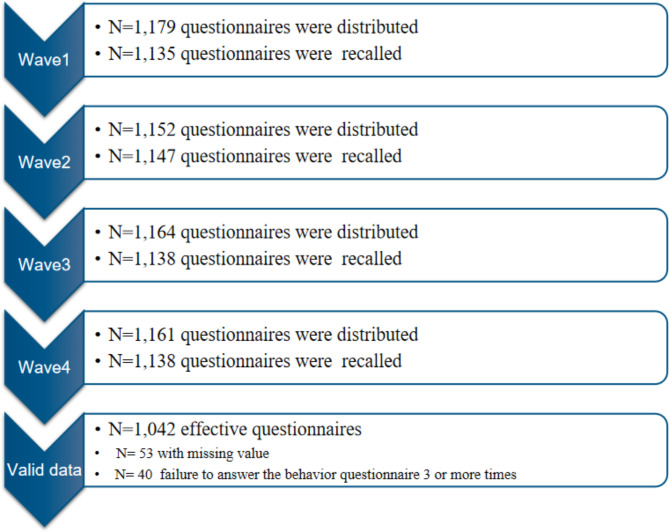
The flow chart of participants

Participants were enrolled in the College Student Behavior and Health Cohort Study, which is an cohort designed to track behaviors, physical and mental health in college students. This research used data from the first four follow-up studies. This cohort was selected from 2 universities in Hefei, Anhui Province and Shangrao, Jiangxi Province in China during April-May 2019, and 1,179 freshmen were selected by cluster random sampling for baseline questionnaire survey. Follow-up surveys were conducted every six months, and by December 2021, a total of three follow-up surveys had been conducted. We needed to fit the trajectories of HRBs, so participants who completed the HRBs questionnaire less than three times were excluded. Finally, 1,042 college students who had both completed the MEQ-5 and behavioral questions over the course of two years. The specific inclusion and exclusion criteria of this study are shown in Fig. 1.

This research was approved by the ethics committee of Anhui Medical University (No.20170291). All participants obtained written informed consent. Figure 1 show the selection criteria for participants.

### Sociodemographic data

Sociodemographic data were collected by the questionnaire, including gender, age, self-reported family economic status, self-reported academic performance, parents’ education level and any siblings.

### Chronotype

During Wave 1, the Morningness-Eveningness Questionnaire-5 (MEQ-5) was used to evaluate the chronotype of college students [22]. The scale consists of 5 items, including wake up time, fatigue time, best time to fall asleep, best state time and self-rated morning/night type, to assess the long-term chronotype of individuals. The total score of the scale ranges from 4 to 25 points. In this research, it was divided into M-type (18 ~ 25 points), N-type (12 ~ 17 points) and E-type (4 ~ 11 points). Cronbach’s α in this study was 0.68.

### Sleep duration

During Wave 1, by asking participants “During the past month, how many hours of actual sleep did you get at night? “. Based on the National Sleep Foundation’s updated sleep duration recommendations [23], students (aged 18 to 25 years) who were sleeping at least 7 h/night, responses were categorized into three groups: short sleep (< 7 h), normal sleep (7–9 h), and long sleep (> 9 h).

### Health-risk behaviors

During Wave 1 ~ Wave 4, the four kinds of HRBs self-reported by college students were evaluated to understand the cumulative health risks.

### Smoking

By asking participants “During the past month, how many days did you smoke cigarettes? “, to judge the smoking status of college students. There are seven options for this question: none, 1–2 days, 3–5 days, 6–9 days, 10–19 days, and almost every day. In this study, the answer “none” means no smoking behavior, and all other options mean smoking behavior.

### Alcohol use

By asking participants “During the past month, how many days have you drunk at least 1 glass of wine? “, to judge the alcohol use status of college students. There are five options for this question: none, 1–2 days, 3–5 days, 6–9 days, or more than 10 days. In this study, the answer “none” means no alcohol use, and all other options mean alcohol use.

### Low physical activity

The physical activity of college students was assessed according to the short version of the International Physical Activity Questionnaire (IPAQ) [24]. The frequency and duration of physical activity of college students in the past week that were less than 600 MET were classified as low physical activity.

### Smartphone addiction

The smartphone addiction of college students was assessed by the Self-rating Questionnaire for Adolescent Problematic Mobile Phone Use (SQAPMPU) [25]. The questionnaire has 13 items, with good reliability and validity, and Cronbach’s α coefficient is 0.87. If the adolescents’ total score was ≥ P75 of the participants, it was determined as smartphone addicts.

### Statistical analysis

SPSS 23.0 was used for data processing and analysis, and the inspection level was α = 0.05.

First, to identify distinct subgroups of participants with different longitudinal trajectories, Mplus version 7.4 was used for the LCGA. This analysis is capable of identifying homogeneous subgroups in a larger heterogeneous population. By running the LCGA model continuously, the number of classifications was increased by one, and the optimal number of classifications was obtained. The model fitting criteria included adjusting the Bayesian information criterion (ABIC), Akaike information criterion (AIC), Bayesian information criterion (BIC), Lo-Mendell likelihood ratio test (LMR), and bootstrap likelihood ratio test (BLRT). The closer the ABIC and BIC values are to zero, the better fitting effect of the model is. The best model should have the minimum ABIC AIC and BIC, and then the combined judgment is based on relative entropy, classification probability, and classification interpret ability. Generally, relative entropy > 0.7 means that the model is in the acceptable range, and the proportion of each classification group should be > 5% of the total population.

Second, the *χ*^2^ test was used to compare the differences between the demographic variables in the trajectories of different HRBs. Ultimately, the multivariate logistics regression model was used to analyze the correlation between the baseline chronotype, sleep duration and the trajectories of HRBs.

## Results

### General information

The 1,042 college students included in the survey had an average age of 18.81 years, of which 654 were females, accounting for 61.9%. More than average students lived in rural areas, and 24.4% of students self-reported that their family economy was poor. Among all students participating in the survey, 77.1% of students reported that their fathers had received junior high school and above education, and 56.1% of students reported that their mothers had received junior high school and above education. In addition, in a total of 1,135 participants, there were no differences between demographic characteristics (age, gender, family economy, academic performance, parental education level) and follow-up loss data at the time of baseline analysis (Table 1).

**Table 1:** The difference of demographic characteristics between analysis data and missing data

Characteristic	Included (*n* = 1042)	Excluded (*n* = 93)	χ^2^/t	*P*-value
Age	18.81 ± 1.18	18.60 ± 0.80	1.52	0.09
Gender			0.01	0.93
Male	397(38.1)	35(37.6)		
Female	645(61.9)	58(62.4)		
Living place				
Rural	583(56.0)	50(53.8)	0.17	0.68
Urban	459(44.0)	43(46.2)		
Any siblings				
Yes	242(23.2)	26(28.0)	1.60	0.30
No	800(76.8)	67(72.0)		
Self-reported family economy				
Low	254(24.4)	19(19.4)	5.78	0.06
Medium	736(70.6)	64(68.8)		
high	52(5.0)	10(11.8)		
Self-reported academic performance			2.60	0.27
Low	220(21.1)	19(20.4)		
Medium	644(61.8)	52(55.9)		
high	178(17.1)	22(23.7)		
Father’s education level			3.79	0.15
Primary school and below	239(22.9)	18(19.4)		
Middle school	500(48.0)	39(41.9)		
Senior high school and above	303(29.1)	36(38.7)		
Mother’s education level			0.77	0.68
Primary school and below	457(43.9)	40(43.0)		
Middle school	366(35.1)	30(32.3)		
Senior high school and above	219(21.0)	23(24.7)		

^a^*P-value* < *0.05.*^b^*P-value* < *0.001.*

### Trajectories of health-risk behaviors

Model fit statistics for models with a varying number of classes are presented in Table 2. Based on the model fitting statistics and the interpret ability of the categories, the best-fitting model was classified into four categories because the BIC values and aBIC values of the four categories were both lows (*P* < 0.05), and the classification probability was ≥ 0.05 at the same time.

**Fig. 2:**
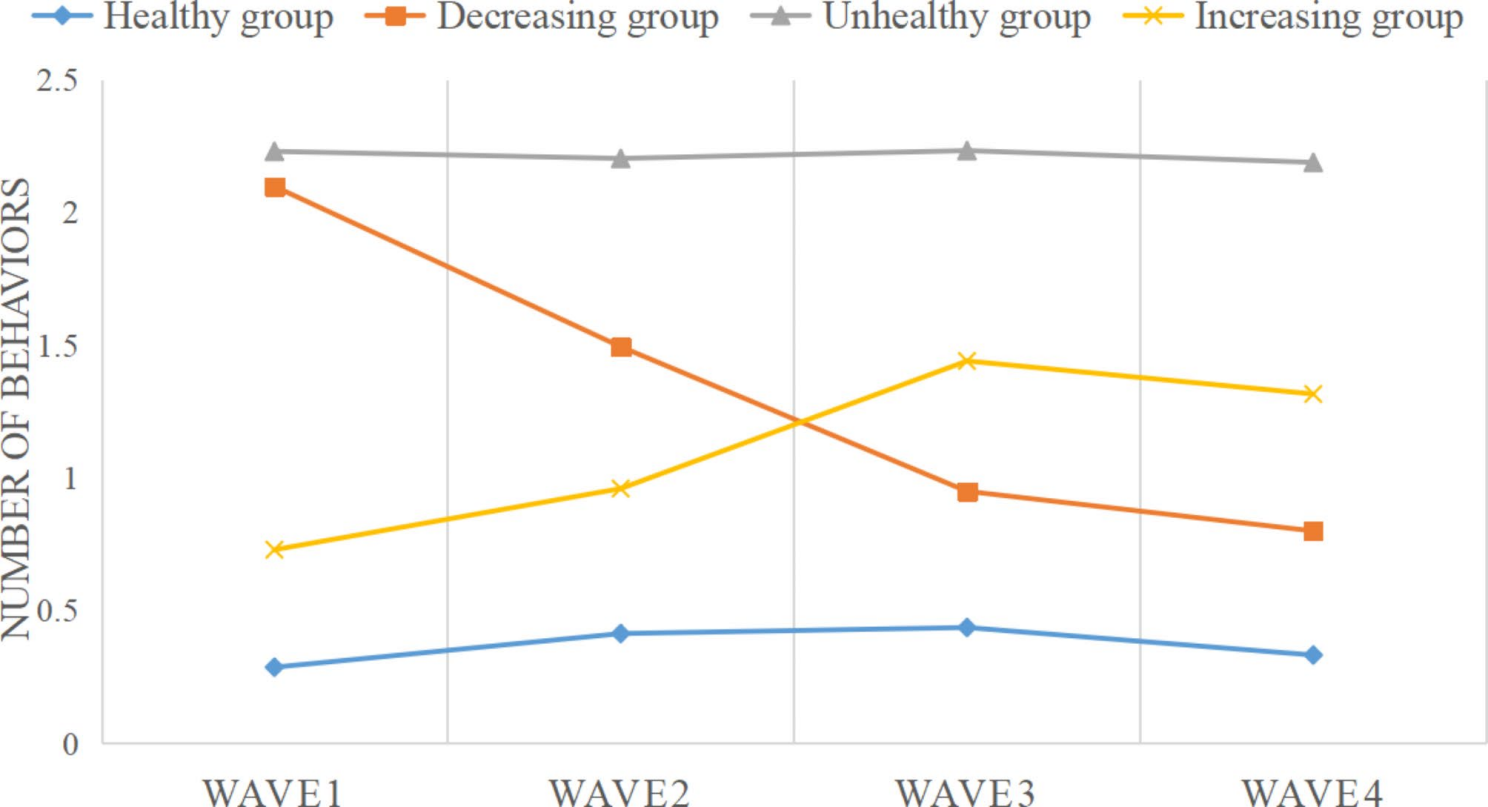
Latent Class Growth Analysis of health-risk behaviors

Figure 2 shows the trajectories of the four latent classes identified. The first trajectory (represented by the diamond marker) is characterized by relatively healthy behaviors, so it is named the healthy group, which accounts for approximately 61.0%. The second trajectory, which accounts for 10.3%, is named the decreasing group. This trajectory shows a gradual decline in HRBs over time. The third trajectory is named the increasing group because it initially rises significantly and then declines slightly over time. 21.3% of college students belong to this group. The last trajectory (represented by the triangle marker) was described as the unhealthy group because individuals following this trajectory had high levels of all four reports. The proportion of people belonging to the unhealthy group is 7.4%.

**Table 2:** Fit indices for latent class growth analysis of health-risk behaviors

Classes	Fit Indices						
	AIC	BIC	ABIC	Entropy	LMR *P*-values	BLRT *P*–values	Classification probability
1 C	10524.35	10558.99	10536.76	1.00	—	—	
2 C	9631.14	9680.63	9648.87	0.86	0.00	0.00	0.20/0.80
3 C	9490.45	9554.78	9513.50	0.74	0.04	0.00	0.30/0.09/0.61
4 C	9367.77	9446.95	9396.13	0.79	0.03	0.00	0.23/0.07/0.60/0.10
5 C	9373.77	9467.79	9407.45	0.82	0.50	0.00	0.23/0.59/0.07/0.10/0.01

AIC is Akaike’s information criterion, BIC is Bayesian information criterion, ABIC is adjusted the Bayesian information criterion, LMR is Lo-Mendell likelihood ratio test, BLRT is bootstrap likelihood ratio test

### Sample characteristics of different health-risk behavior trajectories

Table 3 displays the characteristics and group differences of 1,042 college students. In the baseline sleep duration, the detection rates of short sleep, normal sleep, and long sleep were 35.5%, 57.2%, and 7.3%, respectively. This research also shows that the proportion of M-type (18.7%) was higher than that of E-type (10.1%) among college students. There were significant differences in the distribution of HRBs detection rate among different gender, father’s education level, self-reported academic performance and family economy. Among them, college students who are male, self-reported poor academic performance, self-reported poor family economy and fathers with lower education level, their health-risk behaviors trajectory tends to be unhealthy group (*P* < 0.05). At the same time, compared with the decreasing group, college students in the increasing group tended to be female, have moderate self-reported academic achievement, medium self-reported family economy, and fathers with lower education levels. In addition, the distribution of health risk behaviors also tended to differ among college students with different sleep characteristics. Among them, E-type and long sleep duration college students, their health risk behavior trajectory is often unhealthy group. At the same time, college students in the increasing group tended to have E-type and short sleep duration compared with those in the decreasing group (*P* < 0.05).

**Table 3:** Participants characteristics of health-risk behaviors trajectory groups

Characteristic	*N*	Health-risk behaviors trajectory groups				χ^2^ value
		Healthy group (*n* = 636)	Decreasing group (*n* = 107)	Increasing group (*n* = 222)	Unhealthy group (*n* = 77)	
Gender						101.22^b^
Male	397(38.1)	189(47.6)	65(16.4)	81(20.4)	62(15.6)	
Female	645(61.9)	447(69.3)	42(6.5)	141(21.9)	15(2.3)	
Living place						2.36
Rural	583(56.0)	351(60.2)	62(10.6)	121(20.8)	49(8.4)	
Urban	459(44.0)	285(62.1)	45(9.8)	101(22.0)	28(6.1)	
Any siblings						2.02
Yes	242(23.2)	141(58.2)	29(12.0)	51(21.1)	21(8.7)	
No	800(76.8)	495(61.9)	78(9.8)	171(21.3)	56(7.0)	
Self-reported academic performance						26.60^b^
Low	220(21.1)	115(52.3)	34(15.5)	48(21.8)	23(10.4)	
Medium	644(61.8)	389(60.4)	64(9.9)	143(22.2)	48(7.5)	
high	178(17.1)	132(74.2)	9(5.0)	31(17.4)	6(3.4)	
Self-reported family economy						12.68^a^
Low	254(24.4)	149(58.7)	29(11.4)	50(19.7)	26(10.2)	
Medium	736(70.6)	451(61.3)	77(10.5)	163(22.1)	45(6.1)	
high	52(5.0)	36(69.2)	1(1.9)	9(17.3)	6(11.6)	
Father’s education level						13.74^a^
Primary school and below	239(22.9)	131(54.8)	25(10.5)	55(23.0)	28(11.7)	
Middle school	500(48.0)	311(62.2)	57(11.4)	106(21.2)	26(5.2)	
Senior high school and above	303(29.1)	194(64.0)	25(8.3)	61(20.1)	23(7.6)	
Mother’s education level						8.49
Primary school and below	457(43.9)	265(58.0)	47(10.3)	112(24.5)	33(7.2)	
Middle school	366(35.1)	231(63.1)	34(9.3)	76(20.8)	25(6.8)	
Senior high school and above	219(21.0)	140(63.9)	26(11.9)	34(15.5)	19(8.7)	
Chronotype						32.89^b^
M-type	195(18.7)	121(62.1)	27(13.8)	35(17.9)	12(6.2)	
N-type	742(71.2)	475(64.0)	60(8.1)	154(20.8)	53(7.1)	
E-type	105(10.1)	40(38.1)	20(19.1)	33(31.4)	12(11.4)	
Sleep duration						54.99^b^
Short sleep	370(35.5)	211(57.0)	36(9.7)	94(25.5)	29(7.8)	
Normal sleep	596(57.2)	391(65.6)	55(9.2)	120(20.1)	30(5.1)	
Long sleep	76(7.3)	34(44.7)	16(21.1)	8(10.5)	18(23.7)	

M-type is the morning type, N-type is neutral type, E-type is evening type

^a^*P-value* < *0.05.*^b^*P-value* < *0.001.*

### Association of chronotype, sleep duration and trajectories of health-risk behaviors

**Fig. 3:**
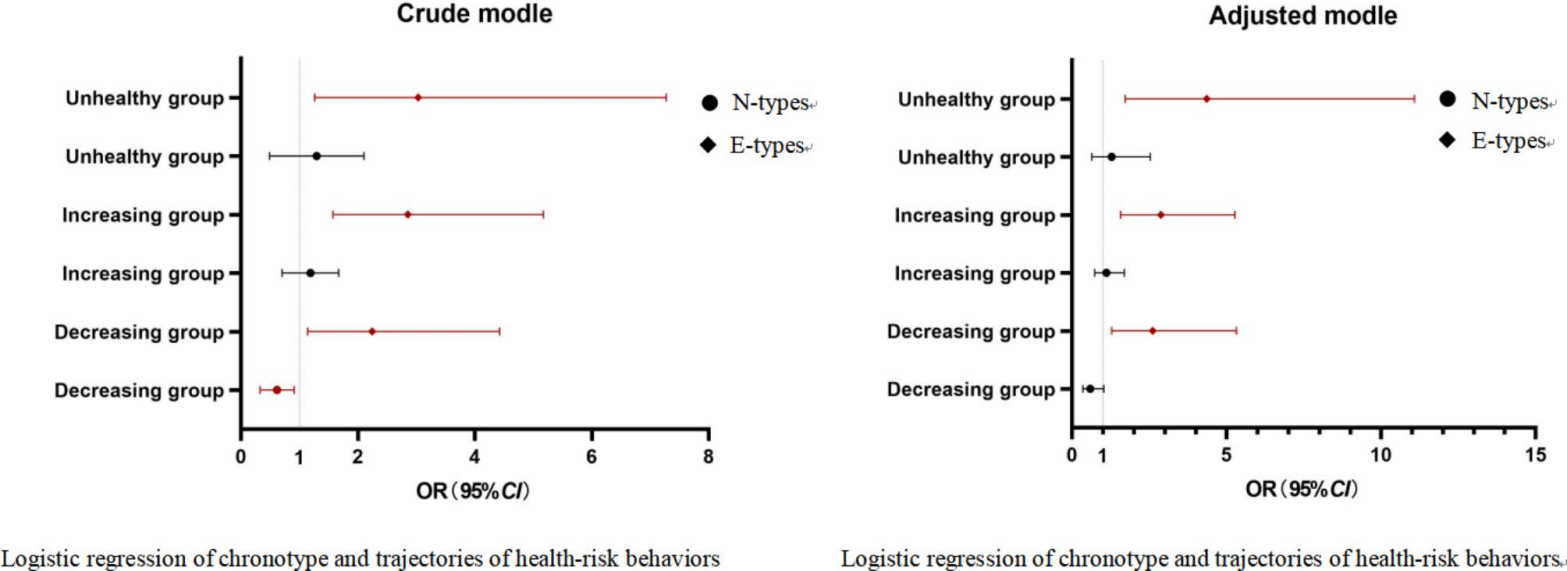
Logistic regression of chronotype, sleep duration and trajectories of health-risk behaviors

Taking healthy group as the reference group, we used multiple logistic regressions to analyse the relationship between sleep parameters and the trajectory of HRBs. Measures of gender, self-reported academic performance, self-reported family economy and father’s education level were included as covariance due to known associations with HRBs. After controlling those covariances, compared with normal sleep, the short sleep was positively correlated with the unhealthy group and the increasing group, the *OR* (95% *CI*) for those were 1.76 (1.01, 3.08), 1.45 (1.05, 1.99), respectively. The long sleep was positively correlated with the unhealthy group, the decreasing group, the *OR* (95% *CI*) for those were 5.82 (2.80, 12.09), 3.06 (1.55, 6.06), respectively. Compared with the M-type, the E-type were positively correlated with the unhealthy group the increasing group, the decreasing group, the *OR* (95% *CI*) for those were 4.36 (1.72, 11.08), 2.88 (1.57, 5.27), 2.61 (1.28, 5.32), respectively (Fig. 3).

## Discussion

To our knowledge, this is the first research to analyze the trajectory of multiple HRBs in college students, and further analyze its relationship between chronotype, sleep duration. Our study found that the trajectories of multiple HRBs over time can be divided into four patterns: healthy group, increasing group, decreasing group and unhealthy group. Short sleep duration and long sleep duration were both associated with high-risk trajectories. The results of the study also identified a significant association between E-type and the trajectory of high-risk trajectories of HRBs.

This research is the first to examine the interdependence trajectories of four HRBs: smoking, alcohol use, low physical activity, smartphone addiction. As expected, the vast majority of people belong to the healthy group (61.0% of sample), while the unhealthy group only account for 7.4% of the total population. Similar percentages of adolescents in high-risk groups were found in the Mobile Youth Study, in which approximately 11% of children aged 12 to 18 exhibited increasing high risk in all three areas, including substance use, conduct problems, and sexual risk taking [26]. However, the study by Zhang et al. through the follow up of 1,974 children aged 7 to 9, the fitting results of four life style (screen time, physical activity, sleep duration and beverage intake) trajectories showed that persistent healthy groups accounted for 39.9%, while 17.7% of children were persistent unhealthy groups [27]. The large differences in grouping may be due to the differences in HRBs included in the studies and the fact that the subjects came from different ages, changing one behavior will affect the prevalence of others [28].

The study found that about 10.1% of college students reported E-type, which was below the 20.5% of students from 8 major Lebanese universities who reported E-types [29]. Compared with M-type, Evening-type was associated with three high-risk HRBs classes. Children are typically M-type, gradually becoming E-type after entering puberty, and teenagers around the age of 20 sleep at the latest [30]. Some previous studies have found that those with later bedtimes had some lifestyle habits that might delay bedtimes, increase depressive symptoms, and lead to health hazards [31]. During adolescence, there is a shift in biologic preference to later sleep timing, which often conflicts with socially prescribed scheduling demands, such as early school start times [32]. It has been speculated that nocturnal time types may affect gene expression, metabolism, immunoendocrine function, and behavior due to the chronic mismatch between internal physiological time and externally imposed time for work and social activities [33]. Previous studies have found that a later phenotype may be more associated with externalized behaviors, such as risk-taking and substance use [34]. Since these behaviors are more likely to occur at night times with less parental supervision, and the E-type provides greater opportunities for participation in such behaviors [35], it should not be surprising that the association with E-type and relatively high-risk trajectories. Furthermore, evening chronotype may contribute to HRBs through impairments in emotion regulation, executive function, response inhibition, and decision making [36, 37].

Of particular note in this research is the association between sleep duration and the trajectory of HRBs. We observed that about 35.5% of students had short sleep, 7.3% of students reported long sleep, which was different from the detection rate of Shandong Adolescent Behavior and Health Cohort [29]. Using data from the Fairfax County Youth Survey, Owens found that students with sleep insufficiency were significantly more likely than respondents without sleep insufficiency to report drug use and delinquency [38]. Based on data from the Project for the Health of Adolescents in Southern Taiwan, the results indicated that both short and long sleep duration were significantly associated with having suicidal and drinking alcohol every week [39]. Previous studies have mainly found the effect of sleep duration on HRBs through cross-sectional studies. However, our research found that not only short but long sleep also affected HRBs trajectories in a cohort study. Regarding the mechanism that leads to the association between sleep duration and HRBs, based on the evidence from a systematic review, suggests that comparing various sleep duration against an “optimal” sleep duration allows non-linear relationships to be observed. U-shaped relationships between sleep duration and risk-taking where both short and long sleep duration are associated with heightened risk-taking [40]. Epidemiological studies have demonstrated that short sleep and sleep disturbance are associated with subsequent impairment in multiple domains of psychosocial and cognitive functioning, delinquency, impulsivity, substance use, and depression [41, 42].

### Strengths and limitations

These findings contribute to the literature is that we use a latent class growth model to fit the trajectory of the HRBs among college students. The combined analysis of relationship between multiple HRBs rather than single HRBs and chronotype, sleep duration is more in line with the needs of real-world research, which can reflect the dynamic changes and influencing factors of HRBs in adolescence. Second, we used a cohort study to carry out a follow-up investigation and collected data on various HRBs of college students many times, which makes the results more convincing. As far as we know, this is the first research to report the association between E-type, short sleep duration and long sleep duration and longitudinal HRBs. The highlight of this research is to explore the influence of circadian rhythm on multiply HRBs, so as to reveal the influence of circadian rhythm on human health.

Despite the above advantages, there is no denying that this research has limitations in the following aspects: First, although we adopted a self-reporting scale that has been fully verified and used to assess sleep characteristics and health risk behaviors among college students, reporting bias is still unavoidable. In addition, this study was conducted in two universities in two provinces of China, the representativeness of the research objects is limited, which leads to the limited extrapolation of the research results. In the future, large-scale cohort studies across the country are still needed for verification.

## Conclusion

Our findings indicated that sleep parameters, including both E-type and short sleep duration and long sleep duration, were common among college students, and they were independently associated with increased odds of HRBs’ trajectory. Insight into the changing patterns and predictors of multiple HRBs in adolescents may contribute to more successful and targeted prevention.

## Data Availability

No datasets were generated or analysed during the current study.

## Abbreviations

HRBs: Health-risk behaviors
MEQ-5: Morning and evening questionnaire-5
LCGA: Latent class growth analysis
M-type: Morning-type
N-type: Neutral -type
E-type: Evening-type
IPAQ: The international physical activity questionnaire
SQAPMPU: The self-rating questionnaire for adolescent problematic mobile phone use
ABIC: Bayesian information criterion
AIC: Akaike information criterion
BIC: Bayesian information criterion
LMR: Lo-Mendell likelihood ratio test
BLRT: bootstrap likelihood ratio test

## References

[1] Forouzanfar MH, Alexander L, Anderson HR, et al. Global, regional, and national comparative risk assessment of 79 behavioural, environmental and occupational, and metabolic risks or clusters of risks in 188 countries, 1990–2013: a systematic analysis for the Global Burden of Disease Study 2013. Lancet. 2015;386:2287–323. 26364544 10.1016/S0140-6736(15)00128-2 PMC4685753

[2] Wijbenga L, de Winter AF, Almansa J, et al. Multiple health risk behaviors and mental health from a life course perspective: the Dutch TRAILS study. Prev Med. 2022;154: 106870. 34780855 10.1016/j.ypmed.2021.106870

[3] De Winter AF, Visser L, Verhulst FC, et al. Longitudinal patterns and predictors of multiple health risk behaviors among adolescents: the TRAILS study. Prev Med. 2016;84:76–82. 26656404 10.1016/j.ypmed.2015.11.028

[4] Noble N, Paul C, Turon H, et al. Which modifiable health risk behaviours are related? A systematic review of the clustering of smoking, nutrition, alcohol and physical activity health risk factors. Prev Med. 2015;81:16–41. 26190368 10.1016/j.ypmed.2015.07.003

[5] Prüss-Ustün A, van Deventer E, Mudu P, et al. Environmental risks and non-communicable diseases. BMJ. 2019;364: l265. 30692085 10.1136/bmj.l265 PMC6348403

[6] Héroux M, Janssen I, Lee DC, et al. Clustering of unhealthy behaviors in the aerobics center longitudinal study. Prev Sci. 2012;13:183–95. 22006293 10.1007/s11121-011-0255-0 PMC3304050

[7] Poortinga W. The prevalence and clustering of four major lifestyle risk factors in an English adult population. Prev Med. 2015;44:124–8. 10.1016/j.ypmed.2006.10.006 17157369

[8] Huang DY, Lanza HI, Murphy DA, et al. Development of risk behaviors adolescence: potential pathways to co-occurrence. Int J Behav Dev. 2012;36:247–57. 24482550 10.1177/0165025412442870 PMC3904442

[9] David Buck, Francesca Frosini. Clustering of unhealthy behaviours over time implications for policy and practice. Available at: https: //www.makingeverycontactcount.org/media/1045/018. Accessed August 2012.

[10] Hautala D, Sittner K, Walls M. Latent trajectories and profiles of commercial cigarette smoking frequency from adolescence to young adulthood among north American indigenous people. Nicotine Tob Res. 2020;22:2066–74. 32270190 10.1093/ntr/ntaa063 PMC7593350

[11] Kuh D, Ben-Shlomo Y, Lynch J, et al. Life course epidemiology. J Epidemiol Community Health. 2003;57:778–83. 14573579 10.1136/jech.57.10.778 PMC1732305

[12] Ge Y, Xin S, Luan D, et al. Association of physical activity, sedentary time, and sleep duration on the health-related quality of life of college students in Northeast China. Health Qual Life Outcomes. 2019;17:124. 31311564 10.1186/s12955-019-1194-x PMC6636029

[13] Montaruli A, Castelli L, Mulè A, et al. Biological rhythm and chronotype: new perspectives in health. Biomolecules. 2021;11:487. 33804974 10.3390/biom11040487 PMC8063933

[14] Park H, Lee HK, Lee K. Chronotype and suicide: the mediating effect of depressive symptoms. Psychiatry Res. 2018;269:316–20. 30172189 10.1016/j.psychres.2018.08.046

[15] Buysse DJ. Sleep health: can we define it? Does it matter? Sleep. 2014;37:9–17. 24470692 10.5665/sleep.3298 PMC3902880

[16] Sirtoli R, Balboa-Castillo T, Fernández-Rodríguez R, et al. The association between alcohol-related problems and sleep quality and duration among college students: a multicountry pooled analysis. Int J Ment Health Addict. 2022;27:1–18. 10.1007/s11469-022-00763-8 PMC8793817 35106062

[17] Becker SP, Jarrett MA, Luebbe AM, et al. Sleep in a large, multi-university sample of college students: sleep problem prevalence, sex differences, and mental health correlates. Sleep Health. 2018;4:174–81. 29555131 10.1016/j.sleh.2018.01.001 PMC5863586

[18] Naja F, Hasan H, Khadem SH, et al. Adherence to the mediterranean diet and its association with sleep quality and chronotype among youth: a cross-sectional study. Front Nutr. 2022;8: 805955. 35127790 10.3389/fnut.2021.805955 PMC8808718

[19] McKnight-Eily LR, Eaton DK, Lowry R, et al. Relationships between hours of sleep and health-risk behaviors in US adolescent students. Prev Med. 2011;53:271–3. 21843548 10.1016/j.ypmed.2011.06.020

[20] Haynie DL, Lewin D, Luk JW, et al. Beyond sleep duration: bidirectional associations among chronotype, social jetlag, and drinking behaviors in a longitudinal sample of US high school students. Sleep. 2018;41:zsx202. 29237053 10.1093/sleep/zsx202 PMC6018914

[21] Mireku MO, Barker MM, Mutz J, et al. Night-time screen based media device use and adolescents’ sleep and health-related quality of life. Environ Int. 2019;124:66–78. 30640131 10.1016/j.envint.2018.11.069

[22] Adan A, Almirall H. Horne & Östberg morningness-eveningness questionnaire: a reduced scale. Pers Individ Diff. 1991;12:241.

[23] Hirshkowitz M, Whiton K, Albert SM, et al. National Sleep Foundation’s updated sleep duration recommendations: final report. Sleep Health. 2015;1:233–43. 29073398 10.1016/j.sleh.2015.10.004

[24] Puciato D, Borysiuk Z, Rozpara M. Quality of life and physical activity in an older working-age population. Clin Interv Aging. 2017;12:1627–34. 29042763 10.2147/CIA.S144045 PMC5634394

[25] Tao S, Fu J, Wang H, et al. The development of self-rating questionnaire for adolescent problematic mobile phone use and the psychometric evaluation in undergraduates. Chin J Sch Health. 2013;34:26–9.

[26] Mustanski B, Byck GR, Dymnicki A, et al. Trajectories of multiple adolescent health risk behaviors in a low-income African American population. Dev Psychopathol. 2013;25:1155–69. 24229555 10.1017/S0954579413000436 PMC4039413

[27] Zhang A, Fang J, Wan Y, et al. Joint trajectories of life style indicators and their links to psychopathological outcomes in the adolescence. BMC Psychiatry. 2021;21:407. 34404392 10.1186/s12888-021-03403-y PMC8369712

[28] Nauha L, Jurvelin H, Ala-Mursula L, et al. Chronotypes and objectively measured physical activity and sedentary time at midlife. Scand J Med Sci Sports. 2020;30:1930–8. 32558967 10.1111/sms.13753

[29] Najem J, Saber M, Aoun C, et al. Prevalence of food addiction and association with stress, sleep quality and chronotype: a cross-sectional survey among university students. Clin Nutr. 2020;39:533–9. 30878156 10.1016/j.clnu.2019.02.038

[30] Randler C, Fassl C, Kalb N. From lark to owl: developmental changes in morningness-eveningness from new-borns to early adulthood. Sci Rep. 2017;7:45874. 28378787 10.1038/srep45874 PMC5381104

[31] Merikanto I, Lahti T, Puusniekka R, et al. Late bedtimes weaken school performance and predispose adolescents to health hazards. Sleep Med. 2013;14:1105–11. 24051113 10.1016/j.sleep.2013.06.009

[32] Carskadon MA, Acebo C, Jenni OG. Regulation of adolescent sleep: implications for behavior. Ann N Y Acad Sci. 2004;1021:276–91. 15251897 10.1196/annals.1308.032

[33] Roenneberg T, Merrow M. The circadian clock and human health. Curr Biol. 2016;26:432–43. 10.1016/j.cub.2016.04.011 27218855

[34] Karan M, Bai S, Almeida DM, et al. Sleep-wake timings in adolescence: chronotype development and associations with adjustment. J Youth Adolesc. 2021;50:628–40. 33606125 10.1007/s10964-021-01407-1 PMC7993411

[35] Randler C, Bilger S. Associations among sleep, chronotype, parental monitoring, and pubertal development among German adolescents. J Psychol. 2009;143:509–20. 19943401 10.3200/JRL.143.5.509-520

[36] Pieters S, Burk WJ, Van der Vorst H, et al. Prospective relationships between sleep problems and substance use, internalizing and externalizing problems. J Youth Adolesc. 2015;44:379–88. 25385390 10.1007/s10964-014-0213-9

[37] Wong MM, Robertson GC, Dyson RB. Prospective relationship between poor sleep and substance-related problems in a national sample of adolescents. Alcohol Clin Exp Res. 2015;39:355–62. 25598438 10.1111/acer.12618 PMC4331208

[38] Owens J, Wang G, Lewin D, et al. Association between short sleep duration and risk behavior factors in middle school students. Sleep. 2017;40:zsw004. 10.1093/sleep/zsw004 28364447

[39] Yen CF, King BH, Tang TC. The association between short and long nocturnal sleep durations and risky behaviours and the moderating factors in Taiwanese adolescents. Psychiatry Res. 2010;179:69–74. 20472300 10.1016/j.psychres.2009.02.016

[40] Short MA, Weber N. Sleep duration and risk-taking in adolescents: a systematic review and meta-analysis. Sleep Med Rev. 2018;41:185–96. 29934128 10.1016/j.smrv.2018.03.006

[41] Kenney SR, Paves AP, Grimaldi EM, et al. Sleep quality and alcohol risk in college students: examining the moderating effects of drinking motives. J Am Coll Health. 2014;62:301–8. 24588270 10.1080/07448481.2014.897953 PMC4031278

[42] Liu X, Liu ZZ, Liu BP, et al. Associations between sleep problems and ADHD symptoms among adolescents: findings from the Shandong adolescent behavior and health cohort (SABHC). Sleep. 2020;43:zsz294. 31790135 10.1093/sleep/zsz294

